# A Transport Vehicle‐enabled TREM2 agonist antibody induces putative transcription factor activation in microglia

**DOI:** 10.1002/alz.090395

**Published:** 2025-01-03

**Authors:** Kathryn M. Monroe

**Affiliations:** ^1^ Denali Therapeutics Inc., South San Francisco, CA USA

## Abstract

**Background:**

Loss‐of‐function variants of TREM2 are associated with increased risk of Alzheimer’s disease (AD), suggesting that activation of this innate immune receptor may be a useful therapeutic strategy. We’ve previously described a high‐affinity mouse TREM2‐activating antibody engineered with a monovalent transferrin receptor (TfR) binding site, termed antibody transport vehicle (ATV), ATV:4D9. Single‐cell RNA sequencing and morphometry revealed that ATV:4D9 shifted microglia to metabolically responsive states, which were distinct from those induced by amyloid pathology. However, the factors mediating ATV:4D9‐induced transcriptional responses in microglia is not known.

**Method:**

We performed fluorescence‐activated nuclei sorting (FANS) to isolate PU.1+ microglial nuclei from frozen brain tissue followed by assaying for transposase‐accessible chromatin with sequencing (ATAC‐Seq) to determine regions of accessible chromatin in the microglial genome.

**Result:**

We identified changes in chromatin accessibility that are observed in microglia dosed with ATV:4D9 compared to isotype control antibody. Bioinformatic analysis identified motifs for putative regulatory transcription factors in ATV:4D9‐activated enhancers.

**Conclusion:**

Our data provide insights into putative transcription factors that mediate microglial state changes in response to ATV:4D9, which further elucidates mechanisms of TREM2 agonism.

